# Physiological Disturbance May Contribute to Neurodegeneration Induced by Isoflurane or Sevoflurane in 14 Day Old Rats

**DOI:** 10.1371/journal.pone.0084622

**Published:** 2014-01-06

**Authors:** Binbin Wu, Zipu Yu, Shan You, Yihu Zheng, Jin Liu, Yajing Gao, Han Lin, Qingquan Lian

**Affiliations:** 1 Department of Anesthesiology, The Second Affiliated Hospital, Wenzhou Medical University, Wenzhou, China; 2 Department of General surgery, The First Affiliated Hospital, Wenzhou Medical University, Wenzhou, China; Imperial College London, Chelsea & Westminster Hospital, United Kingdom

## Abstract

**Background:**

Volatile anesthetics are widely used in pediatric anesthesia but their potential neurotoxicity raise significant concerns regarding sequelae after anesthesia. However, whether physiological disturbance during anesthetic exposure contributes to such side effects remains unknown. The aim of the current study is to compare the neurotoxic effects of isoflurane and sevoflurane in 14 day old rat pups under spontaneous breathing or ventilated conditions.

**Methods:**

Postnatal 14 day rats were assigned to one of five groups: 1) spontaneous breathing (SB) + room air (control, n = 17); 2) SB + isoflurane (n = 35); 3) SB + sevoflurane (n = 37); 4) mechanical ventilation (MV) + isoflurane (n = 29); 5) MV + sevoflurane (n = 32). Anesthetized animal received either 1.7% isoflurane or 2.4% seveoflurane for 4 hours. Arterial blood gases and blood pressure were monitored in the anesthetized groups. Neurodegeneration in the CA3 region of hippocampus was assessed with terminal deoxynucleotidyl transferase-mediated DNA nick-end labeling immediately after exposure. Spatial learning and memory were evaluated with the Morris water maze in other cohorts 14 days after experiments.

**Results:**

Most rats in the SB groups developed physiological disturbance whereas ventilated rats did not but become hyperglycemic. Mortality from anesthesia in the SB groups was significantly higher than that in the MV groups. Cell death in the SB but not MV groups was significantly higher than controls. SB + anesthesia groups performed worse on the Morris water maze behavioral test, but no deficits were found in the MV group compared with the controls.

**Conclusions:**

These findings could suggest that physiological disturbance induced by isoflurane or sevoflurane anesthesia may also contribute to their neurotoxicity.

## Introduction

Volatile anesthetics such as isoflurane [1] and sevoflurane [2] likely act on gamma aminobutyric acid (GABA_A_) receptors that have been demonstrated to be neurotoxic in the developing brain. Brain growth rate is highest during the first 2 postnatal weeks in rodents [3], [4]. Previous studies indicated long-term exposure to volatile anesthetics during this period cause neuronal death in the hippocampus and subsequently lead to cognitive dysfunction [5], although other studies have reported conflicting results [6], [7]. These findings raise concerns regarding the long-lasting neurological sequelae of anesthesia-exposure during early childhood. Many studies, however, were carried out without airway management, animals were placed in an anesthetic chamber flushed continuously with volatile anesthetics mixed in air and pure oxygen, in which mortality reached up to 20–30% and blood gases were often beyond the physiological ranges including metabolic acidosis [8]–[10]. However, whether those physiological disturbance caused by anesthetic induced states contributes to the neurotoxicity is open a question. Herein we report a comparison study to determine whether there were the different changes of physiological parameters, mortality, neurotoxicity and cognitive function when postnatal 14 day rats were anesthetized with isoflurane or sevoflurane for 4 hours under either spontaneous breathing or mechanical ventilation.

## Methods

### Ethics Statement and Animals

All experiments were approved by the Animal Care and Use Committee of Wenzhou Medical University without a number (Wenzhou, China), and all surgery was performed under anesthesia, and all efforts were made to minimize animal use including that the sample size was calculated with 90% power at a significance level of 0.05 according to the data obtained from preliminary experiments. Animals were housed under a 12/12 h light-dark cycle at 22–24°C and given free access to food and water. Male, postnatal day 14, Sprague–Dawley rats (n = 150) were randomly assigned to control (n = 17), spontaneously breathing anesthetized (n = 72) and mechanically ventilated anesthetized groups (n = 61). Both anesthesia-treated groups were subdivided into 1.7% isoflurane or 2.4% sevoflurane treatment groups. Spontaneously breathing groups were treated in a sealed transparent plastic chamber, as described previously (spontaneously breathing group, isoflurane n = 35, sevoflurane n = 37). Mechanically ventilated groups were treated using an anesthesia machine (ALC-V, animal ventilator, Alcott, Biotech, China; VMR, animal anesthesia machine, Matrx, USA; isoflurane n = 29, sevoflurane n = 32).

### Spontaneously Breathing and Anesthetized with Isoflurane or Sevoflurane

Rat pups in the spontaneously breathing groups were placed in plastic anesthesia chambers equipped with a gas inlet and outlet on each end and two holes in the top cover to accommodate the connections for gas concentration monitoring, blood pressure monitoring and blood sampling for blood gas analysis. Chambers were flushed continuously with either 1.7% isoflurane (Baxter Healthcare Corporation., New Providence, USA) (IS group) or 2.4% sevoflurane (Abbott Laboratories, North Chicago, IL 60064, USA) (SS group) in a mixture of air and pure oxygen (1∶1) for 4 hours at 2 L/min. The temperature of the chamber floor was kept at 37°C with a computer-controlled heating/cooling plate (SS20-2, ZH0017022, Huaibei, China) integrated into the floor. Rectal temperature was monitored and maintained at 36.5±0.5°C. To avoid carbon dioxide rebreathing, the chamber floor was covered with 1 cm depth of soda lime. The concentration of isoflurane or sevoflurane was continuously monitored with a gas analyzer (ARYM-0054 Vamos, Dräger, Germany). Rats were continuously monitored during experiments and any mortality was recorded.

### Mechanical Ventilation with Isoflurane or Sevoflurane

Rats in the mechanically ventilated groups were induced with 2% isoflurane (IM group) or 3% sevoflurane (SM group) in a special induction box. After successful induction, the anterior part of rats was lightened and the rats were orotracheally intubated with a 20-gauge catheter (IntroCan®-W, B/Braun, Melsungen, Germany) under direct visualization of the glottis. Then the catheter was then connected to the anesthesia ventilator and animals were treated for 4 hours with either 1.7% isoflurane or 2.4% sevoflurane under a volume controlled mode (V_T_: 5ml, rate: 35 times/minute, I:E = 1∶2). Ventilation parameters were determined in pilot studies in which the blood gases were kept within the normal physiological ranges. Mixed gases were administered at a flow rate of 2 L/min. Rectal temperature was maintained at 36.5±0.5°C and any mortality and time of death were recorded during experiments.

### Blood Gas Analysis and Arterial Pressure Measurements

Arterial blood (0.3 ml) was sampled from three rats in each group of the four anesthetized groups via inserting 24-gauge polyethylene catheters (IntroCan®-W, B/Braun) through the abdominal aorta. Insertion was conducted using a dissecting microscope (PS100, Nikon, Tokyo, Japan). Rats in the mechanical ventilation groups were ventilated constantly to insure proper anesthesia depth when exposing the abdominal aorta. In the spontaneously breathing groups, the membrane sealed hole was opened and the rats body was pulled outside the chamber for arterial blood pressure monitoring and blood gas analysis. The rats head was kept within the chamber throughout the procedure, and the voids around the head were sealed with a membrane. Mean arterial blood pressure (MAP) was detected with a measuring instrument (M3046A, Philips, Boeblingen, Germany). Blood samples were immediately analyzed to determine pH, arterial oxygen, carbon dioxide and blood glucose values (GEM Premier 3000, Bedford, MA, USA). Rats in the control group were anesthetized with ketamine for blood gas measurement (10 mg⋅kg^−1^, i.p. injection; Fujian, China, n = 3).

### TUNEL Staining

After recovering from anesthesia, 6 rats in each group were deeply anesthetized and perfused transcardiacally with chilled 0.9% normal saline followed by 4% paraformaldehyde in phosphate buffered saline (PBS) (0.1 M, pH 7.4). Their brains were removed and kept in 4% paraformaldehyde in phosphate buffered saline overnight at 4°C and then embedded in paraffin, which was cut into 5 µm sections with a microtome (RM2235, Leica, Wetzlar, Germany). TUNEL staining was performed according to the manufacturer’s instructions (Roche, Indianapolis, IN, USA). The number of TUNEL positive cells was counted using an inverted microscope (80iS/N 551364, Nikon) in the CA3 region of the hippocampus. Six random fields were counted in each section at ×400 magnification in a blinded manner. The percentages of TUNEL immunopositive cells were calculated for each group.

### Behavioral Observations and Neurocognitive Testing

Spatial learning and memory were assessed in the Morris water maze beginning at 2 weeks after anesthesia exposure. The Morris water maze had a diameter of 150 cm and was kept at room temperature (24°C) in a quiet room with dim light. Distant and visual cues were hung on the walls around the pool, and the animals searched for a 12 cm diameter platform submerged 1 cm below the surface of water. The rats underwent four trials each day in the pool at four different starting positions following a predetermined 5 day sequence. Each trial was conducted for up to 120 s to allow the rats time to find the platform with a 15 s intertrial interval. Rats that failed to find the platform in the allotted time were guided onto the platform. The distance (pathlength) and time (escape latency) to reach the platform was automatically recorded (SLY-WMS Morris water maze, Shuolinyuan, Beijing, China). On day 6, a probe trial was conducted, in which the platform was moved and animals were introduced into the quadrant opposite the initial platform position. Rats were allowed 60 s to search the tank. The time spent in the quadrant that the platform was previously located in was recorded (target quadrant dwell time).

### Statistical Analysis

Data are presented as mean ± standard deviation. Blood gas analysis and probe trial data were compared using one-way analysis of variance (ANOVA). The Morris water maze data were analyzed with repeated-measures ANOVA with Dunnett’s *post hoc* test for multiple comparisons. MAP data in the four anesthetized groups were analyzed with one-way ANOVA and the LSD *post hoc* test for multiple comparisons. Nonparametric data and the percentage of TUNEL immunopositive cells were analyzed using the Kruskal–Wallis test in combination with Dunn’s *post hoc* analysis for multiple comparisons. Anesthesia-induced mortality was analyzed using a χ2 contingency table analysis. Statistical calculations were performed with SPSS 17.0. A p<0.05 was considered to be significant.

## Results

### Anesthesia Induces Physiological Disturbance in the Spontaneously Breathing Groups but not in the Mechanically Ventilated Groups

Fourteen-day-old rats from each group were anesthetized and arterial blood was sampled. Table 1 summarizes the physiological data at the end of each hour. After 1 hour of anesthesia, the average PaCO2 in the SS group was increased by 40 mmHg relative to the control group (p = 0.006). PaCO2 did not increase in the IS group (p = 0.857). Correspondingly, pH and PaO2 in the SS group decreased significantly (pH, p = 0.002, PaO2, p = 0.034). After 3 hour of anesthesia, PaCO2 in the IS and SS groups were higher, whist pH and PaO2 were lower relative to controls. No significant difference in pH and PaCO2 was found between the mechanically ventilated and control groups (p>0.05), but PaO2 in both ventilated groups was higher than controls because of inspiration of 50% oxygen (p<0.05). These results indicate that rats in both spontaneously breathing groups experienced several hours of hypercapnia and acidosis during the experiment. Moreover, a glucose metabolism disorder developed during anesthesia, as blood glucose was significantly higher in all anesthetized groups. At the end of the first hour, MAP of rats in the spontaneously breathing groups was not remarkably different from that in the mechanically ventilated groups (IS vs. IM p = 0.65, SS vs. SM p = 0.455), whereas MAP of rats in the IS and SS groups was significantly lower than that in the IM and SM groups in the following hours, respectively (IS vs. IM, 2 h p = 0.001, 3 h p = 0.041, 4 h p = 0.018; SS vs. SM, 2 h p = 0.012, 3 h p = 0.023, 4 h p = 0.022).

**Table 1:** Physiologic data in 14-day -old rats exposed to 1.7% isoflurane or 2.4% sevoflurane by either spontaneous breathing or mechanical ventilation at the end of each hour during 4-hour anesthesia.

Parameters				Control			IS				SS	
					1 h	2 h	3 h	4 h	1 h	2 h	3 h	4 h
pH				7.37±0.04	7.20±0.09	7.13±0.15*	7.03±0.07**	7.06±0.14**	7.22±0.05*	7.12±0.10*	7.19±0.11*	7.10±0.06**
PaCO2	(mm Hg)			46.5±6.2	58.0±17.1	89.0±21.3*	99.0±10.8*	94.0±18.2*	86.0±10.5*	81.3±25.1*	77.3±9.6*	95.7±6.7*
PO2	(mm Hg)			91.3±2.2	68.7±6.6*	63.4±2.8*	70.3±12.7 *	64.7±4.5*	68.7±8.3*	68.3±8.1*	70.0±2.6*	68.5±4.5*
Glu	(mg/dl)			109.9±11.5	137.9±35.3	189.2±10.0*	151.3±23.6	169.9±31.5*	174.1±12.0*	148.3±47.3	172.9±39.0*	185.0±19.4*
MAP	(mm Hg)				52±3	48±3**	46±3*	46±2*	50±3	47±3*	46±2*	44±2*
**Parameters**				**Control**			**IM**				**SM**	
					**1 h**	**2 h**	**3 h**	**4 h**	**1 h**	**2 h**	**3 h**	**4 h**
pH				7.37±0.04	7.44±0.02	7.37±0.03	7.39±0.02	7.41±0.06	7.45±0.04	7.38±0.04	7.34±0.06	7.38±0.05
PaCO2	(mm Hg)			46.5±6.2	44.0±6.0	45.3±1.2	43.0±5.3	36.0±1.0	44.6±6.8	43.7±5.1	50.3±4.2	45.3±6.4
PO2	(mm Hg)			91.3±2.2	171.7±8.3*	195.7±15.5*	193.0±11.1*	207.7±35.0*	196.3±10.6*	191.0±12.5*	196.7±3.8**	209.0±6.1
Glu	(mg/dl)			109.9±11.5	173.5±7.2*	174.2±22.7*	179.3±24.5*	138.8±49.4*	152.9±33.9*	166.1±23.3*	174.8±20.5*	190.1±11.9
MAP	(mm Hg)				53±3	61±4	55±6	54±5	52±2	55±3	56±5	52±3

Data presented as mean±standard deviation.

IS = Isoflurane spontaneously breathing group; SS = Sevoflurane spontaneously breathing group;

IM = Isoflurane mechanically ventilated group; SM = Sevoflurane mechanically ventilated group.

pH, PaCO2, PO2 and Glu: *P<0.05, **P<0.01, compared with controls.

MAP: *P<0.05, **P<0.01, IS compared with IM, SS compared with SM.

### Mortality in the Spontaneously Breathing Groups was Significantly Higher than the Mechanically Ventilated Groups

We intubated 61 rats, 6 rats were excluded because of injuring the trachea or piercing the throat, 2 rats in the IM group and 4 in the SM group. Besides, 1 rat died in the IM group and 2 in the SM group because the catheter was blocked by secretions during anesthesia. Thus, we successfully intubated 55 rats, and mortality in the IM and SM groups was 3.7% and 7.1%, which was caused by the anesthetics themselves was 0(data not shown). Other 52 rats were ventilated for 4 hours, 26 rats with 1.7% isoflurane and 26 rats with 2.4% sevoflurane. All ventilated rats recovered from anesthesia rapidly, and no cyanopathy or twitch occurred after extubation, so the recovery rate was 100%. Nine and 11 rats died during anesthesia in the IS and SS groups, respectively. Mortality in the IS group was 25.7% and in the IM group was 29.7%. Mortality was significantly higher in the spontaneously breathing groups relative to the ventilated groups (Table 2, IS vs. IM, p = 0.033; SS vs. SM, p = 0.030). Death occurred mainly in the last 2 hours of the experiments.

**Table 2:** Mortality due to anesthesia exposure in the spontaneously breathing groups and the mechanically ventilated groups.

Charactertistics	IS	SS	IM	SM
1 h	0	1	0	0
2 h	2	2	0	0
3 h	2	4	0	2
4 h	5	4	1	0
The number of rats died	9	11	1	2
Total number of ratsused	35	37	27	28
Mortality (%)	25.7*	29.7*	3.7	7.1

IS = Isoflurane spontaneously breathing group;

SS = Sevoflurane spontaneously breathing group;

IM = Isoflurane mechanically ventilated group;

SM = Sevoflurane mechanically ventilated group.

Anesthesia mortality in isoflurane and sevoflurane spontaneously breathing groups was significantly higher than isoflurane and sevoflurane mechanically ventilated groups, respectively.

p<0.05.

### Cell Death was Significant Higher in the Spontaneously Breathing Groups, but not in the Control or Ventilated Groups

**Figure 1:**
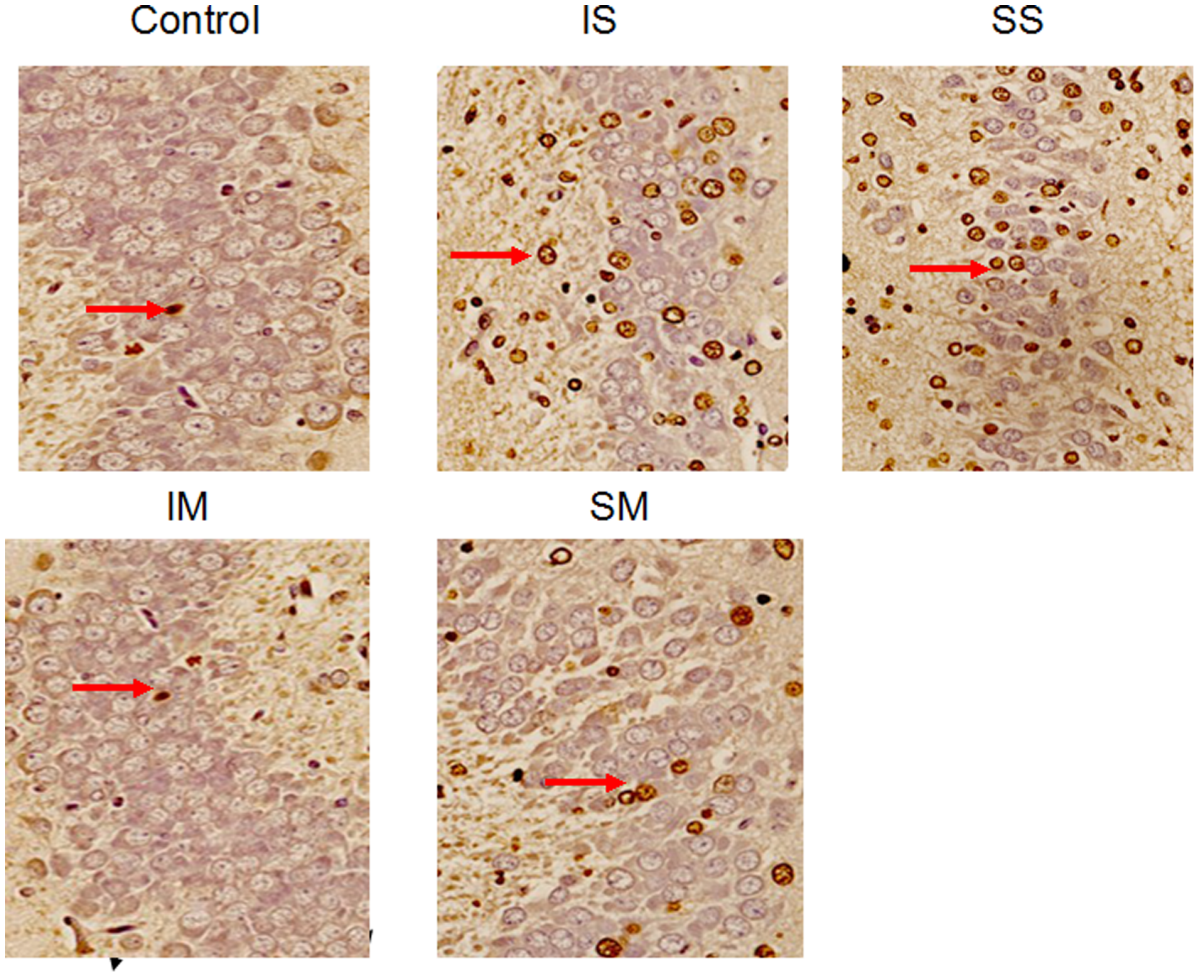
Images of the CA3 region in the hippocampus (5 µm) of five groups, the expression of the terminal deoxynucleotidyl transferase-mediated DNA nick-end labeling (TUNEL) apoptosis marker. Less cell death in the control and mechanically ventilated groups compared with the spontaneously breathing groups. Positively stained cells are chocolate brown, and arrows mark representative TUNEL-positive cells in each group. IS, isoflurane spontaneously breathing group; SS, sevoflurane spontaneously breathing group; IM, isoflurane mechanically ventilated group; SM, sevoflurane mechanically ventilated group.

**Figure 2:**
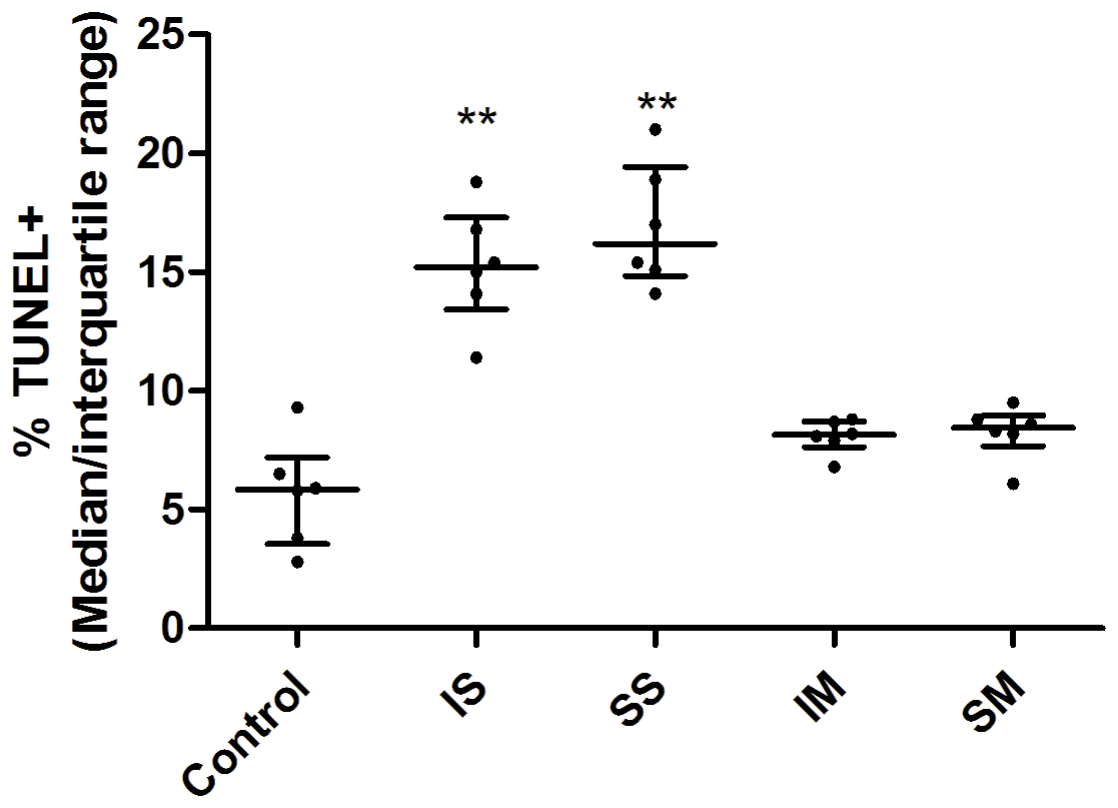
More cell death in the spontaneously breathing groups. Hippocampal cells stained positive for the early apoptosis TUNEL marker immediately after 4-hour anesthesia. Neonatal isoflurane and sevoflurane exposure increased terminal deoxynucleotidyl transferase-mediated DNA nick-end labeling (TUNEL) expression in the spontaneously breathing groups. The results indicate a significant difference compared with the control, but no statistical difference between the isoflurane or sevoflurane mechanically ventilated groups and control group. **p<0.01.

Six rats from each group were used to quantify cell death with TUNEL staining. The CA3 region of the hippocampus, which is important for memory and learning [11], was examined. Cells were counted and the percentage of TUNEL immunopositive cells was determined (Fig. 1). Results showed that the percentages of TUNEL immunopositive cells in the IS and SS groups were significantly higher than those in the control group (Fig. 2, p<0.01). There was no significant difference between isoflurane and sevoflurane treated groups (p = 0.378). Although the TUNEL immunopositive cells in the IM and SM groups were more than in the control group, no significant difference was found among these three groups (Fig. 2, p = 0.085).

### Behavioral Observations and Neurocognitive Testing

**Figure 3:**
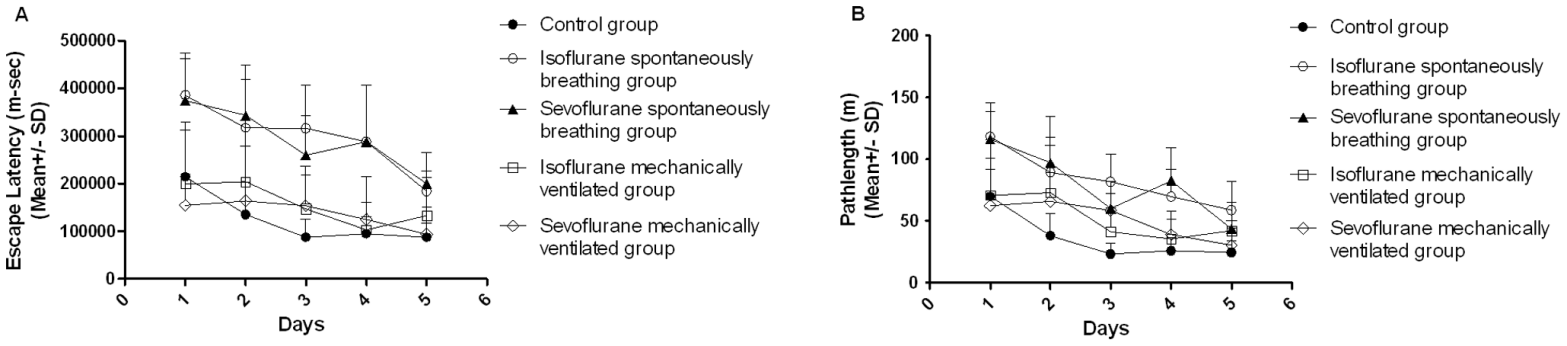
Rats in the spontaneously breathing groups performed worse than other groups when searching platform. Time to reach the platform (escape latency, A) and the distance to reach the platform (pathlength, B) were recorded. Data points represent the average of the sum of four daily trials from four different quadrants for 5-day hidden platform trials. Error bars represent the standard deviation. Although all groups improved over the 5-day trial period, A shows that the rats exposed to 1.7% isoflurane or 2.4% sevoflurane in the chamber took more time to search for the platform than those of the control (p<0.001, repeated-measures ANOVA), but no difference was found among the control and mechanically ventilated groups. Similarly, B shows pathlength was much longer in the spontaneously breathing groups than in the control group (p<0.001, repeated-measure ANOVA). No significant difference was observed among the control and mechanically ventilated groups. (p>0.05).

**Figure 4:**
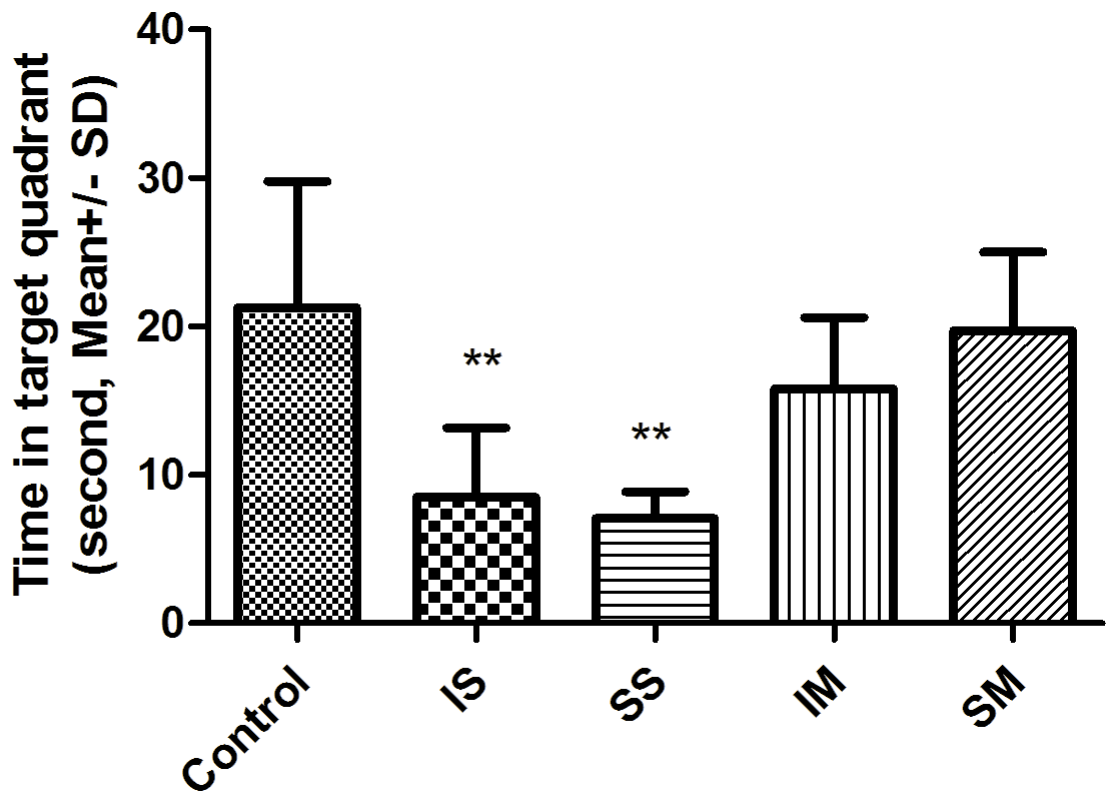
Rats in the spontaneously breathing groups performed worse than other groups in spatial probe testing. Time spent by each group in the quadrant with the removed platform of each group is shown. Rats exposed to 1.7% isoflurane and 2.4% sevoflurane in chambers did impair short-term spatial learning, the rats spent much less time in the target quadrant than the controls. But no difference between the control group and the isoflurane, or sevoflurane mechanically ventilated group.

Eight rats from each group underwent behavioral testing 2 weeks after anesthesia exposure. Escape latency and pathlength decreased in all groups during the 5 day period, indicating that rats learned the location of the platform. Rats in the IS and SS groups, however, performed more poorly than the control group, and the ventilated isoflurane and sevoflurane groups. Both escape latency and pathlength were significantly longer (Fig. 3, p<0.001). By contrast, although escape latency and pathlength in the IM and SM groups tended to be longer compared to controls, the differences were not significant. During the probe trial (day 6), animals in the IS and SS groups spent significantly less time in the target quadrant when compared with the control group (Fig. 4, p<0.01). Rats in the ventilated groups also tended to spend less time in the target quadrant, but the effect was not significant. Finally, within the spontaneously breathing and mechanically ventilated groups, no difference was found between isoflurane and sevoflurane. Thus, it may be concluded that spatial learning and reference memory were impaired in rats from the spontaneously breathing groups but not in the mechanically ventilated groups.

## Discussion

In mammals, neurogenesis, differentiation, migration, medullation and cell death in the central nervous system are simultaneously occurring during the all involved in neurodevelopment stage, and learning and memory are mostly associated with the hippocampus, particularly the CA3 region [12]. An impaired hippocampus which is the brain structure that is mostly associated with learning and memory would cause apparent spatial cognitive dysfunction during this stage. The hippocampal CA3 area locates in the center of the crosstalks of massive inputs, reported to be crucial for information retention and relate closely with spatial learning consolidation process [11], [13]. Indeed, exposing newborn rats to anesthetics during this sensitive periods may induce neurotoxicity and massive neuronal cell death, leading to impaired memory and neurocognitive ability. Many studies have reported isoflurane and sevoflurane induced widespread cell death and memory impairment *in vitro*, and *in vivo* [10], [14], [15]. For example, sevoflurane exposure caused significant neurodegeneration and spatial reference memory deficits in postnatal 7 day rats at concentrations of 3–5% [16], and exposure with the same concentration of isoflurane resulted in increased brain cell degeneration in mice, but did not cause significant behavioral deficits [17]. Other studies indicated that cell death caused by isoflurane and sevoflurane had no relationship with functional memory impairment [17]. Furthermore, postnatal 6 day rats exposed to 3–5% sevoflurane for 4 hours did not show significant cell death or spatial memory learning deficit as assessed by Morris water maze testing [18]. Therefore, there are some uncertainties to make a conclusion from previous studies as stated above. We cannot equate anesthetics induced cell death to cognitive dysfunction at present. The discrepancy may result from different age subjects, different doses, test timing, and species but how homeostasis in those tiny animals during experiments has not been investigated intensively due to technical difficulties, or others.

The mechanism of anesthetics induce cell death is not known well, but some studies have suggested the apoptotic cell death might not be induced by isoflurane itself rather than a physiological byproduct [19]–[21]. Hypercapnia and respiratory or metabolic acidosis all could contribute to cell death and high mortality [7]–[9]. Skin color changed and diminished respiration in animals during anesthesia were observed in dead or even in surviving rats [19], suggesting that respiratory insufficiency and metabolic abnormalities occurred even in rats that survived from anesthesia. This phenomenon also occurred in this study. It was also reported 4-hour hypercapnia treatment would cause widespread brain cell death but not spatial reference memory deficits, which might be attributed to different age subjects and test timing [9].

Given the limitations of previous anesthesia exposure methods, pneumothorax caused by cardiac puncture that affects arterial blood gas analysis, especially in newborn rodents, failure in collecting arterial blood from carotid artery, and excessive blood loss and hypothermia caused by monitoring abdominal aortic pressure continuously, we exposed 14-day-old rats to anesthetics by mechanical ventilation for 4 hours with interval MAP measurement and blood gas analysis to make sure no hypoxia, hypercapnia, acidosis or hypotension happened during the experiments throughout. To reach a balance between volatile anesthetics concentrations and end-tidal levels, the anesthetics were delivered with 2 L/min fresh gas. MAP was 50–65 mmHg during anesthesia in both the IM and SM groups, but was decreased in the spontaneously breathing groups. Blood gas analysis indicated serious hypercapnia and acidosis occurred and continued for several hours, the decreased MAP and metabolic derangement might constitute a vicious circle. Mortality was significantly higher in the spontaneously breathing groups than that in the mechanically ventilated groups. TUNEL immunopositive cells in the CA3 region of the hippocampus, an early expression of cell death, were more intensive in both spontaneously breathing groups than the control group, and rats in the two groups performed worse on Morris water maze testing. No significant behavioral deficits were found among the mechanically ventilated and control groups. These findings suggest differences among the anesthetized groups may be attributed to respiratory and circulation derangement, but not caused by the anesthetics themselves mainly. Nevertheless, we cannot exclude the possibility of subtle deficits caused by isoflurane and sevoflurane in the mechanically ventilated groups, which were not detected by Morris water maze testing in this study. Learning and memory deficits may appear if it was performed earlier or later, or with other behavioral tests. Although isoflurane was reported to induce significant apoptosis in postnatal 6 day old rhesus macaque brain by 5-hour anesthesia with endotracheal intubation, mechanical ventilation and intense physiologic monitoring, we attribute the discrepancy to different species, age subjects, doses and test timing [22].

The definition of hypoglycemia varies among different medical centers, such as 40 mg⋅dL^−1^ [23] or 60 mg⋅dL^−1^ [20]. Based on this, no rat in this study was hypoglycemic, but hyperglycemia developed in all anesthetized groups. A similar result was reported by Zhu [10], who exposed postnatal 14 day rats to 1.7% isoflurane, but some other studies reported isoflurane caused hypoglycemia [7], [24]. The differences may be from different species, anesthesia doses, or duration. Enflurane [25], isoflurane [26], and sevoflurane [27] may impair glucose tolerance by decreasing insulin secretion and glucose utilization [28], and activating of K-ATP channels [29], [30]. Moreover, this effect of isoflurane or sevoflurane on blood glucose was equal, and did not vary as altering inhaled concentration [31]. Regardless of the degree of hyperglycemia, it should not be responsible for neurodegeneration or anesthesia related mortality, because it occurred in all anesthetized rats. MAC changes with age, we presumed 1.7% isoflurane and 2.4% sevoflurane in postnatal 14 day rats was about 1.0 MAC according to the results of Orliaquet [32]. Anesthetics cause an intrinsic cerebral vasodilatory effect in a dose-dependent manner [33], [34]. One or 2 MAC of isoflurane and sevoflurane was demonstrated to increase mean cerebral blood flow (CBF) by autoradiography, compared with controls [35]. Furthermore, Lu demonstrated autoregulation of CBF was not impaired during 1 MAC of sevoflurane anesthesia in rats, although MAP decreased to some extent [36]. Thus, we considered the concentrations that we used in our experiments would not cause cerebral insufficiency or autoregulatory impairment, cell death and learning deficits were likely caused by physiological byproducts of anesthetics in the spontaneously breathing groups, at least at those concentrations used in our experiments.

Neurogenesis reaches its peak on postnatal 7 day in rodents, and several studies published adopted that old rats, we also chose postnatal 14 day rats because of high intubation failure rate in smaller rats and considering postnatal 14 day rats were also in critical period of brain maturation. There is a paucity of information about the behavioral impact of anesthesia on postnatal 14 day, Jevtovic-Todorovic showed 25 mg⋅kg^−1^ propofol treatment would not induce neurodegeneration in postnatal 14 day rats even kept them ventilating spontaneously. We have treated postnatal 14 day rats with 40 mg⋅kg^−1^ propofol abdominally without intubation, finding no respiratory depression or physiological derangement occurred (data not published), but 1.7% isoflurane and 2.4% sevoflurane would, indicating these anesthetics effected differently on respiratory depression, despite of this, we also found anesthetics did not cause neurodegeneration, which was consistent with the result of Jevtovic-Todorovic [37]. In addition, Stratmann demonstrated isoflurane caused cell death and cognitive dysfunction [9], we attribute the discrepancy to different ages, because a critical factor for neurodegeneration from anesthesia may be the age of neurons but not the animals, so the effect of anesthetics may be different between newborn neurons of postnatal 7 day rats and 7-day-old neurons of postnatal 14 day rats.

Data from animals far from being used for human, it works differently in anesthesia management between animal studies and clinical pediatric anesthesia practice [19], [38], and this topic has been emphasized in the forum of “Pediatric Anesthesia and Neurodevelopment Assessment” held in 2012–was the cell death caused by anesthesia or physiologically? Did we adopt a most proper anesthesia model to imitate the clinical general anesthesia? For these reasons, we strengthened airway management for the rats in the mechanically ventilated groups to exclude the influence from physiological derangement, which is of significance for studying the toxicity of anesthetics, even for other studies when the animals needs to be operated with anesthesia. However, several limitations of our research should be mentioned. Firstly, despite the fact that concentrations of anesthetics were detected with a monitor, the end-tidal concentration was unknown because the tidal volume was too little to be detected. Secondly, we only compared the cell death in CA3 sector, but cortex and other sectors in the hippocampus were also important for behavior. Thirdly, the Morris water maze testing was carried out only at 2 weeks after anesthesia; thus, we assessed the impact of physiological derangement on young rats within a short period, the long-term influence remained unknown. Although significant difference was found, we did not do other behavioral testings for different fields of cognitive function. Finally, 7-day-old rodents were used in the most published work except mentioned above rather than those 14-day-old used in our study. Therefore, using our data to indicate that anesthetic induced neuroapoptosis in those studies are also due to their physiological effects are likely wrong. However, our work reported here may call that physiological disturbance induced by anesthetics contributing to their neurotoxicity should not be neglected.

## Conclusions

We exposed postnatal 14 day rats to 1.7% isoflurane and 2.4% sevoflurane using two anesthesia methods: one exposed rats in closed chambers, and the other involved mechanical ventilation. We did not find hypercapnia or acidosis in the mechanically ventilated groups but in the spontaneously breathing groups. Significantly increased immunopositive TUNEL expression and learning deficit were detected in the spontaneously breathing groups, whereas no differences were found among the mechanically ventilated and control groups. Thus, cell death and neurocognitive impairment might result from physiological byproducts of isoflurane and sevoflurane under our experimental conditions but may be not due to their coherent toxicity.
