# Correction: Physiological Disturbance May Contribute to Neurodegeneration Induced by Isoflurane or Sevoflurane in 14 Day Old Rats

**DOI:** 10.1371/annotation/e95c286b-92a9-4c02-81b7-90f339dc9e42

**Published:** 2014-01-13

**Authors:** Binbin Wu, Zipu Yu, Shan You, Yihu Zheng, Jin Liu, Yajing Gao, Han Lin, Qingquan Lian

In the Funding statement, the name of a funding organization is incorrect. The Funding statement should read: 

"The work was supported in part by National Science Foundation of China (81271469) and Research Foundation of Doctor Program (20113321110003) to QL, Natural Science Foundation of Zhejiang Province (Z2101211) to QL. The funder is the corresponding author of this study, and he gave final approval of the version to be published."

