# Correction: Physiological Disturbance May Contribute to Neurodegeneration Induced by Isoflurane or Sevoflurane in 14 Day Old Rats

**DOI:** 10.1371/journal.pone.0091207

**Published:** 2014-03-17

**Authors:** The PLOS ONE Staff

The first author, Binbin Wu, is incorrectly indicated in the byline as a corresponding author. In addition, the seventh author, Han Lin, should be indicated as a corresponding author. Han Lin's email address for the purposes of correspondence regarding the article is: nanlinhannansh@qq.com.

In the Funding statement, the name of a funding organization is incorrect. The Funding statement should read: "The work was supported in part by National Science Foundation of China (81271469) and Research Foundation of Doctor Program (20113321110003) to QL, Natural Science Foundation of Zhejiang Province (Z2101211) to QL. The funder is the corresponding author of this study, and he gave final approval of the version to be published."
